# Factors associated with loss to follow-up in retinopathy of prematurity screening referrals at a South African tertiary hospital: A retrospective cohort study

**DOI:** 10.4102/jcmsa.v4i1.430

**Published:** 2026-09-12

**Authors:** Masego Madibane, Carl-Heinz Kruse, Jerusha S. John

**Affiliations:** 1 Discipline of Ophthalmology, College of Health Sciences, University of KwaZulu-Natal, Durban, South Africa; 2 Ophthalmology, Private Practice, Westville Eye Hospital, Durban, South Africa

**Keywords:** retinopathy of prematurity, loss to follow-up, screening, preterm infant, referral pathway, neonatal care, South Africa, KwaZulu-Natal

## Abstract

**Background:**

Continuity of retinopathy of prematurity (ROP) screening follow-up is essential, as the disease progression can occur after the initial examination. Evidence on factors associated with loss to follow-up (LTFU) in South African ROP services is limited.

**Methods:**

This retrospective cohort study reviewed 1698 infants referred for ROP screening at Inkosi Albert Luthuli Central Hospital from 2016 to 2021. Demographic and clinical data were extracted from referral records and the electronic medical records. Loss to follow-up was defined as failure to attend any scheduled follow-up after the initial visit. Baseline (first-visit) characteristics were analysed separately from post-baseline follow-up descriptors. Univariable and multivariable logistic regression identified factors associated with LTFU.

**Results:**

Among the infants, 457 (26.9%) were LTFU. LTFU was more common among infants with lower birth weight, lower gestational age, anaemia, transfusion history, maternal HIV exposure and documented ROP. In multivariable analysis, documented ROP remained strongly associated with LTFU (adjusted odds ratio [aOR] 2.06; 95% confidence interval [CI] 1.60–2.67; *p* < 0.001). Maternal HIV exposure was also independently associated with LTFU in a sensitivity model (aOR 1.58; 95% CI 1.13–2.22; *p* = 0.008).

**Conclusion:**

More than one in four referred infants did not complete scheduled ROP screening follow-up, with those most in need of surveillance being most vulnerable to interrupted care. Strengthened counselling, clearer inter-facility handover and active reminder or tracing systems may improve continuity of care.

**Contribution:**

This study provides local evidence on factors associated with incomplete ROP follow-up in a South African tertiary referral pathway and identifies practical targets for service improvement.

## Introduction

Retinopathy of prematurity (ROP) is a vasoproliferative disorder of the developing retina in preterm infants and remains an important cause of preventable childhood visual impairment and blindness.^1^ Although survival of very preterm and very low birth weight infants has improved in many regions, that progress has been accompanied by continued demand for timely ROP screening, repeated surveillance examinations, and rapid treatment when disease progresses.^2,3,4,5^

The contemporary burden of ROP is particularly relevant in low- and middle-income countries (LMICs), where neonatal intensive care has expanded faster than specialised ophthalmic services.^6,7^ Recent global and regional analyses show that ROP remains common, with the burden increasingly concentrated in settings where screening pathways, transport systems and specialist workforce capacity are uneven.^8^ In Africa, emerging evidence confirms that ROP is no longer rare and that programmes must respond to both improved preterm survival and persistent service constraints.^9,10,11^

Follow-up is especially important in ROP, as retinal vascularisation is often incomplete at the first examination and clinically significant disease may develop or worsen after discharge from the neonatal unit.^12,13^ South African guidelines have long emphasised screening schedules that continue until the retina is adequately vascularised or treatment has been completed. The effectiveness of these recommendations depends on reliable attendance at each planned review.^11,14^ Recent South African reports have highlighted gaps in programme coverage, referral completion, and treatment pathways, reinforcing concern that avoidable blindness can still occur when infants do not return timeously.^10,11,15^

In practice, the transition from inpatient neonatal care to outpatient ophthalmic follow-up is a particularly vulnerable point in the ROP care pathway. Infants are often discharged while still requiring serial retinal examinations, and caregivers may need to navigate complex instructions, repeated appointments and transport challenges while managing the broader medical needs of a premature infant.^16,17,18^ Where booking systems, counselling and communication between facilities are inconsistent, even infants who are appropriately identified for screening may fail to receive the continuity of care needed to prevent avoidable visual loss.^16,19^

A growing international literature suggests that non-attendance is shaped by both clinical and contextual factors, including disease severity, discharge timing, socioeconomic deprivation, caregiver understanding, transport barriers, and the quality of coordination between neonatal and ophthalmic teams.^16,17,18,19,20^ Locally relevant evidence on which infants are most vulnerable to loss to follow-up (LTFU) after ROP screening referral remains limited. This study, therefore, aimed to determine the proportion of infants who did not return for scheduled ROP screening follow-up visits at a tertiary hospital in KwaZulu-Natal and to identify factors associated with interrupted follow-up.

## Research methods and design

### Study design

This was an observational retrospective cohort study of infants referred for ROP screening from 2016 to 2021.

### Setting

The study was conducted at Inkosi Albert Luthuli Central Hospital (IALCH), a public quaternary referral hospital in Durban, KwaZulu-Natal, South Africa. Inkosi Albert Luthuli Central Hospital receives ROP referrals from state health facilities across the eThekwini metropolitan area and wider province and uses an integrated electronic medical record (EMR) system.

### Care pathway and cohort definition

Infants were referred into the tertiary ROP screening service from neonatal units at provincial facilities or were already admitted at IALCH and attended an initial referral or screening visit. They were then expected to return for scheduled surveillance examinations until retinal vascularisation was complete, disease had regressed, or treatment and post-treatment monitoring had concluded. Infants entered the cohort at their first attended visit at IALCH. Follow-up time was calculated as the interval between the first visit date and the last recorded visit date in the EMR.

### Study population and sampling strategy

All infants aged 0–12 months referred to IALCH for ROP screening during the study period were considered eligible. Consecutive referrals recorded in the EMR and accompanying referral documentation were reviewed. A total of 1711 referral records were identified, with 5 excluded due to missing date of first visit and 8 excluded due to implausible birth data. The final analytic sample included 1698 infants. Records with implausible birth data were defined as birth weight values below 500 g or above 2500 g and gestational age values below 20 weeks or above 45 weeks.

### Data collection

Screening was conducted in accordance with South African ROP guidelines.^14^ Demographic and clinical information were extracted from referral letters and EMR entries. Variables included referring hospital, sex, birth weight, gestational age, age at first visit, oxygen therapy, sepsis, anaemia, neonatal jaundice, transfusion history, maternal human immunodeficiency virus (HIV) exposure, infant HIV polymerase chain reaction (PCR) status, ROP status and treatment history.

### Outcome definition

The primary outcome was LTFU, defined operationally as the absence of any attended scheduled follow-up visit in the EMR after the initial referral visit, in an infant for whom screening had not been recorded as complete (full retinal vascularisation, disease regression or completion of treatment and post-treatment monitoring). One missed scheduled visit was considered sufficient for classification as LTFU only when no subsequent attended ROP visit, transfer documentation or discharge from screening was recorded in the available EMR by the end of data extraction. Infants who returned later than scheduled but attended a subsequent visit and had a documented pathway endpoint were not classified as LTFU. This outcome therefore identifies infants who did not attend any scheduled follow-up after the initial visit or did not complete screening.

### Definition of retinopathy of prematurity status

Retinopathy of prematurity status reflected whether ROP was documented at any examination during the infant’s screening episode at IALCH. The dataset did not reliably distinguish ROP documented at the first examination from ROP documented later during surveillance. Detection at any visit depends partly on the number of examinations attended, so associations with ROP status were interpreted as associations with documented disease during available observation rather than as evidence that first-visit ROP caused later non-attendance.

### Baseline versus post baseline variables

Variables recorded at or before the first ROP visit (sex, birth weight, birth gestational age, postnatal and gestational age at first visit, referral source and neonatal comorbidities) were treated as baseline explanatory variables. Variables determined during the follow-up period, namely follow-up duration and gestational age at last visit, were treated as post-baseline descriptors and were not entered as explanatory variables in the regression models, because they are consequences of remaining in care and partly encode the outcome.

### Data analysis

Categorical variables were summarised using frequencies and percentages, and continuous variables using means and standard deviations. Group comparisons were performed using chi-square tests for categorical variables and *t*-tests or Mann-Whitney U tests for continuous variables. The outcome variable was binary, defined as whether an infant was lost to follow-up or remained in care and was therefore analysed using univariable and multivariable logistic regression. Although dates of first and last visit were available, scheduled follow-up dates, missed visit dates and a consistent grace period were not reliably recorded in the EMR, making a valid time-to-event analysis of disengagement not possible.

The adjusted model included prespecified baseline covariates with acceptable completeness: sex, postnatal age at first visit, referral source, oxygen therapy, birth weight category, birth gestational age category, gestational age at first visit, neonatal jaundice, anaemia, transfusion and ROP status. Infant HIV PCR status was recorded for only 171 infants and was excluded from the adjusted model, because its missingness would have restricted the complete case sample to approximately 151 infants and produced unstable estimates. Maternal HIV exposure was examined in a separate sensitivity model. Missingness for each covariate was examined; the analytic sample size and number of LTFU events for the adjusted model are reported, and collinearity was assessed using variance inflation factors. Statistical significance was set at *p* < 0.05. Analyses were performed in Stata 18 (StataCorp LLC, College Station, TX, United States).

### Ethical considerations

Ethical clearance to conduct this study was obtained from the University of KwaZulu-Natal and the Biomedical Research Ethics Committee (No. BREC/00009222/2025), with permission from the gatekeeper, IALCH. The study used retrospective routine clinical data. There was no direct patient contact and informed consent was waived. Confidentiality was protected by anonymising the dataset and storing the linking document separately from the research data.

## Results

A total of 1698 infants were included in the analysis. Of these, 457 (26.9%) were classified as LTFU and 1241 (73.1%) attended at least one scheduled follow-up visit after referral. Table 1 summarises the baseline categorical characteristics of the participants.

**TABLE 1:** Baseline demographic and clinical characteristics of participants (categorical variables; *N* = 1698).

Variables	All patients		LTFU			
			No		Yes	
	*n*	%	*n*	%	*n*	%
**Total**	1698	100	1241	73.1	457	26.9
**Sex (*n* = 1697)**						
Female	849	50.0	629	50.7	220	48.1
Male	848	50.0	611	49.3	237	51.9
**PNA at 1st visit**						
< 4 weeks	150	8.8	115	9.3	35	7.7
4–6 weeks	1055	62.2	776	62.5	279	61.1
> 6 weeks	4934	29.0	350	28.2	143	31.3
**Referral Hospital**						
Clairwood	70	4.1	54	4.4	16	3.5
IALCH	70	4.1	45	3.6	25	5.5
VMH	446	26.3	328	26.4	118	25.8
KDHC	55	3.2	44	3.5	11	2.4
MGMH	57	3.4	38	3.1	19	4.2
Other	118	6.9	90	7.3	28	6.1
RKKH	411	24.3	297	23.9	114	24.9
SMH	92	5.4	59	4.7	33	7.2
Unknown	379	22.34	286	23.0	93	20.4
**Maternal HIV exposure (*n* = 820)**						
No	438	54.4	337	57.6	101	43.0
Yes	382	46.6	248	42.4	134	57.0
**Infant HIV PCR positive (*n* = 171)**						
No	166	97.1	111	98.2	55	94.8
Yes	5	2.9	2	1.8	3	5.2
**Oxygen therapy (*n* = 1488)**						
No	174	11.7	136	12.5	38	9.4
Yes	1314	88.3	949	87.54	365	90.6
**Birthweight (*n* = 1683)**						
< 1000 g	267	15.9	177	14.4	90	19.8
1000 g – 1499 g	1100	65.4	792	64.4	308	67.8
≥ 1500 g	316	18.8	260	21.2	56	12.3
**Birth GA (*n* = 1696)**						
< 28 weeks	158	9.3	101	8.2	57	12.5
28–31 weeks	1026	60.5	724	58.4	302	66.1
≥ 32 weeks	512	30.2	422	33.4	98	21.4
**GA at 1st visit (*n* = 1696)**						
< 32 weeks	65	3.8	44	3.6	21	4.6
32–34 weeks	317	18.7	209	16.8	108	23.6
> 34 weeks	1314	77.5	986	79.6	328	71.8
**Neonatal jaundice**						
No	1263	74.4	912	73.5	351	76.8
Yes	435	25.6	329	26.5	106	23.2
**Anaemia**						
No	1418	83.5	1052	84.8	366	80.1
Yes	280	16.54	189	15.2	91	19.9
**Any transfusion**						
No	1377	81.1	1028	82.8	349	76.4
Yes	321	18.9	213	17.2	108	23.6
**Sepsis**						
No	1213	71.4	890	71.7	323	70.7
Yes	485	28.6	351	28.3	134	29.3
**ROP present**						
No	1011	59.5	809	65.24	202	44.2
Yes	687	40.5	432	34.8	255	55.8

GA, gestational age; HIV, human immunodeficiency virus; IALCH, Inkosi Albert Luthuli Central Hospital; VMH, Victoria Mxenge Hospital; KDHC, King Dinuzulu Hospital Complex; LTFU, loss to follow-up; MGMH, Mahatma Gandhi Memorial Hospital; PCR, polymerase chain reaction; PNA, postnatal age; RKKH, R.K. Khan Hospital; ROP, retinopathy of prematurity; SMH, St Mary’s Hospital.

The sex distribution was similar between groups, with females comprising 50.7% of infants in non-LTFU and 48.1% in the LTFU. Most infants presented for their first visit at 4–6 weeks postnatal age in both groups, 62.5% among those retained and 61.1% among those LTFU. Presentation after 6 weeks was slightly more common among infants who were LTFU, 31.3% compared with 28.2% among those retained. Oxygen therapy was common in both groups. Neonatal jaundice and sepsis were similarly distributed.

Maternal HIV exposure was more frequent among infants who were LTFU than among those retained in care, 57.0% compared with 42.4%. Anaemia and any transfusion history were also more common in the LTFU group, occurring in 19.9% and 23.6% of infants, respectively, compared with 15.2% and 17.2% among those retained. Infant HIV PCR positivity was uncommon overall and proportionally higher among infants who were LTFU, 5.2% compared with 1.8%.

The birth profile differed between groups. Birthweight below 1000 g was more common in the LTFU group, 19.8% compared with 14.4% among those retained. Birth at less than 28 weeks’ gestation was also more frequent among infants who were LTFU, 12.5% compared with 8.2%. Birthweight of at least 1500 g and birth at 32 weeks’ gestation or later were less common among infants who were LTFU. Documented ROP was more frequent in the LTFU group, 55.8% compared with 34.8%. Non-attendance was therefore more common among infants with greater clinical and ophthalmic vulnerability.

Table 2 summarises the clinical variables of the participants and demonstrates a similar pattern to that observed in the categorical analysis. Post baseline descriptors (gestational age at last visit and follow-up time) are reported separately and were not used as explanatory variables.

**TABLE 2:** Demographic and clinical characteristics of participants (continuous variables; *N* = 1698).

Variables	All patients		LTFU			
			No		Yes	
	Mean	s.d.	Mean	s.d.	Mean	s.d.
Baseline						
Birth GA (weeks)	30	2.3	31	2.3	30	2.1
Birth weight (g)	1262.4	271.11	1283.2	273.7	1206	255.9
GA at 1st visit (weeks)	36.46	9.60	36.83	11.10	35	2.5
PNA at 1st visit (weeks)	6	9.5	6.4	11.1	6	2.1
Days on oxygen	13	17.2	12.6	17.2	14	17
Post baseline (descriptive only)						
GA at last visit (weeks)	42	13.9	43	15.8	40	5.7
Follow-up time (weeks)	6	13.5	7	15.3	4	5.2

Note: Gestational age at last visit and follow-up time are determined during the follow-up period and are reported descriptively only; they were not entered as explanatory variables.

GA, gestational age; LTFU, loss to follow-up; PNA, postnatal age; s.d., standard deviation.

Compared with infants who remained in follow-up, those in the LTFU group had a slightly lower mean birth gestational age of 30.1 weeks compared with 31 weeks, and a lower mean birth weight of 1206 g and 1283 g. The mean gestational age at first visit was also lower among infants who were LTFU, 35 weeks compared with 37 weeks among those retained. The mean postnatal age at first visit was similar between groups, 6 weeks in both groups. Duration of oxygen therapy was also similar, with a mean of 14 days among infants who were LTFU and 13 days among those retained.

Table 3 presents the logistic regression analysis of factors associated with LTFU.

**TABLE 3:** Factors associated with loss to follow-up in retinopathy of prematurity screening (univariate and adjusted).

Variables	Univariable			Primary adjustment			Sensitivity adjusted		
	uOR	95% CI	*p*-value	aOR	95% CI	*p*-value	aOR	95% CI	*p*-value
**Sex**									
Female (reference)	1	-	-	1	-	-	1	-	-
Male	1.11	0.89–1.37	0.345	0. 13	0.89–1.44	0.306	0.90	0.64–1.27	0.560
**PNA at 1st visit**									
< 4 weeks (reference)	1	-	-	1	-	-	-	-	-
4–6 weeks	1.18	0.79–1.77	0.4174	1.31	0.80–2.15	0.290	0.99	0.51–1.88	0.964
> 6 weeks	1.34	0.88–2.05	0.175	1.234	0.69–2.20	0.478	0.77	0.35–1.67	0.507
**Referral hospital**									
Clairwood (reference)	1	-	-	1	-	-	1	-	-
IALCH	1.88	0.89–3.94	0.097	1.15	0.50–2.640	0.73	1.32	0.55–3.19	0.532
VMH	1.21	0.66–2.19	0.524	0.71	0.37–1.38	0.312	0.78	0.38–1.61	0.499
KDHC	0.84	0.35–2.00	0.700	0.41	0.15–1.07	0.068	0.48	0.11–2.18	0.342
MGMH	1.69	0.77–3.69	0.191	0.98	0.41–2.30	0.955	0.86	0.32–2.29	0.767
Other	1.05	0.52–2.11	0.891	0.632	0.29–1.36	0.233	0.74	0.30–1.86	0.526
RKKH	1.30	0.70–2.32	0.396	0.91	0.47–1.76	0.773	0.89	0.42–1.87	0.751
SMH	1.89	0.93–3.80	0.076	1.27	0.58–2.79	0.554	1.36	0.52–3.61	0.531
Unknown	1.10	0.59–1.98	0.763	0.80	0.41–1.58	0.527	0.62	0.26–1.44	0.266
**Oxygen therapy**									
No (reference)	1	-	-	1	-	-	1	-	-
Yes	1.38	0.95–2.03	0.099	1.00	0.67–1.50	0.998	1.00	0.57–1.77	0.990
**Birth weight**									
< 1000 g (reference)	1	-	-	1	-	-	1	-	-
1000 g – 1499 g	0.76	0.57–1.01	0.066	1.08	0.76–1.54	0.656	1.05	0.63–1.75	0.845
≥ 1500 g	0.42	0.27–0.60	< 0.001	0.73	0.45–1.19	0.206	0.72	0.36–1.42	0.338
**Birth GA**									
< 28 weeks (reference)	1	-	-	1	-	-	1	-	-
28–31 weeks	0.76	0.52–1.05	0.092	0.84	0.52–1.37	0.492	0.54	0.27–1.09	0.084
≥ 32 weeks	0.42	0.27–0.61	< 0.001	0.64	0.35–1.17	0.146	0.33	0.14–0.78	0.012
**GA at 1st visit**									
< 32 weeks (reference)	1	-	-	1	-	-	1	-	-
32–34 weeks	1.08	0.61–1.91	0.784	1.22	0.62–2.41	0.571	2.56	1.01–6.50	0.048
> 34 weeks	0.70	0.40–1.17	0.186	1.04	0.51–2.14	0.90	2.67	1.00–7.10	0.049
**Neonatal jaundice**									
No (reference)	1	-	-	1	-	-	1	-	-
Yes	0.84	0.65–1.07	0.1654	0.90	0.66–1.230	0.511	0.94	0.65–1.37	0.749
**Anaemia**									
No (reference)	1	-	-	1	-	-	1	-	-
Yes	1.38	1.05–1.83	0.021	0.24	0.47–1.45	0.506	0.79	0.37–1.68	0.537
**Any transfusion**									
No (reference)	1	-	-	1	-	-	1	-	-
Yes	1.49	1.15–1.95	0.003	1.42	0.86–2.33	0.170	1.34	0.67–2.70	0.412
**Sepsis**									
No (reference)	1	-	-	1	-	-	1	-	-
Yes	1.05	0.83–1.33	0.675	1.03	0.74–1.42	0.878	0.98	0.65–1.48	0.927
**ROP present**									
No (reference)	1	-	-	1	-	-	1	-	-
Yes	2.36	1.91–2.96	< 0.001	9.06	1.60–2.67	< 0.001	1.94	1.34–2.80	< 0.001
**Maternal HIV exposure**									
No (reference)	-	-	-	-	-	-	-	-	-
Yes	1.80	1.33–2.45	< 0.001	-	-	-	1.58	1.13–2.22	0.008

aOR, adjusted odds ratio; CI, confidence interval; GA, gestational age; HIV, human immunodeficiency virus; LTFU, loss to follow-up; PNA, postnatal age; ROP, retinopathy of prematurity; uOR, unadjusted odds ratio; IALCH, Inkosi Albert Luthuli Central Hospital; VMH, Victoria Mxenge Hospital; KDHC, King Dinuzulu Hospital Complex; MGMH, Mahatma Gandhi Memorial Hospital; PCR, polymerase chain reaction; PNA, postnatal age; RKKH, R.K. Khan Hospital; SMH, St Mary’s Hospital.

In the univariable analysis, maternal HIV exposure (unadjusted odds ratio [uOR] 1.80; 95% confidence interval [CI] 0.33–2.45; *p* < 0.001), anaemia (uOR 1.38; 95% CI 1.05–1.83; *p* = 0.021), transfusion history (uOR 1.49; 95% CI 1.15–1.95; *p* = 0.003), documented ROP (uOR 2.36; 95% CI 1.90–2.94; *p* < 0.001), lower birth weight and lower birth gestational age were associated with increased odds of LTFU. By contrast, birth weight ≥ 1500 g (uOR 0.41; 95% CI 0.27–0.60; *p* < 0.001) and birth gestational age ≥ 32 weeks (uOR 0.41; 95% CI 0.27–0.61; *p* < 0.001) were associated with lower odds of LTFU. Lower birth weight and lower birth gestational age were also associated with increased odds of LTFU: relative to a birth weight below 1000 g, the odds were progressively lower at 1000 g – 1499 g (uOR 0.76; 95% CI 0.57–1.02; *p* = 0.066) and at ≥ 1500 g (uOR 0.42; 95% CI 0.29–0.62; *p* < 0.001); and relative to a birth gestational age below 28 weeks, the odds were lower at 28–31 weeks (uOR 0.74; 95% CI 0.52–1.05; *p* = 0.092) and at ≥ 32 weeks (uOR 0.42; 95% CI 0.28–0.62; *p* < 0.001).

In the multivariable analysis, documented ROP remained independently and robustly associated with LTFU (adjusted odds ratio [aOR] 2.06; 95% CI 1.60–2.67; *p* < 0.001). No other covariate was independently associated with LTFU in the primary model. In a sensitivity model that also included maternal HIV, documented ROP remained associated with LTFU (aOR 1.94; 95% CI 1.34–2.80; *p* < 0.001) and maternal HIV exposure was independently associated with LTFU (aOR 1.58; 95% CI 1.13–2.22; *p* = 0.008). Birth gestational age ≥ 32 weeks (aOR 0.33; 95% CI 0.14–0.78; *p* = 0.012) and the gestational age at first visit categories (32–34 weeks aOR 2.56; 95% CI 1.01–6.50; *p* = 0.048; > 34 weeks aOR 2.67; 95% CI 1.00–7.10; *p* = 0.049) reached significance; these estimates were imprecise, with wide CIs reflecting sparse cells, and should be interpreted with caution. Collinearity among covariates was acceptable (maximum variance inflation factor 7.2, observed among the correlated birth weight, birth gestational age and gestational age at first visit terms; mean 3.4).

Figure 1 shows the difference in follow-up status according to ROP diagnosis. Among infants who completed follow-up, most had no documented ROP (65.2%), whereas among infants who were LTFU, a greater proportion had documented ROP (55.8%) than did not (44.2%). This was statistically significant (*p* < 0.001) and is consistent with both the categorical analysis and the adjusted model.

**FIGURE 1:**
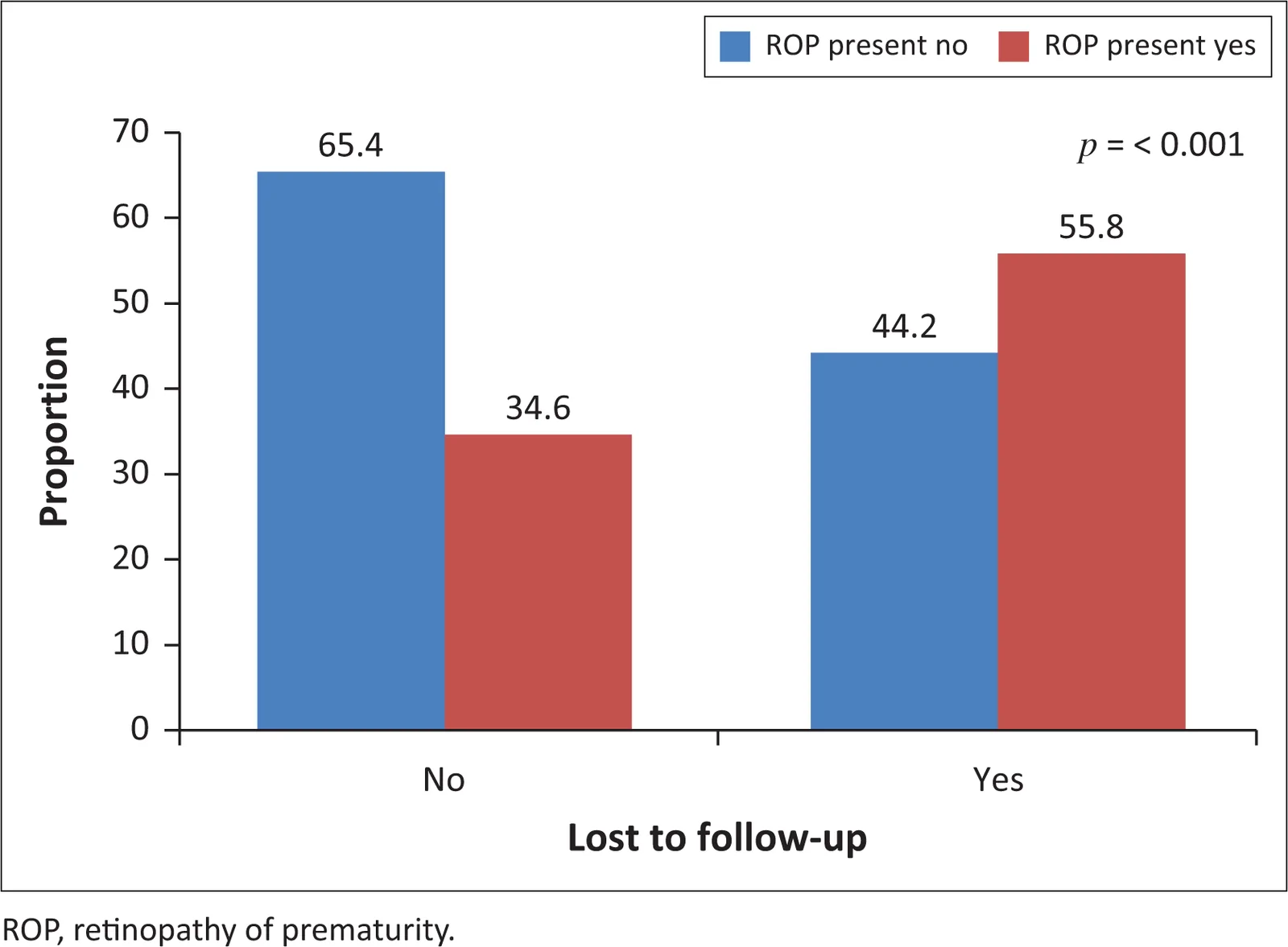
Proportions of infants by loss to follow-up status and presence of retinopathy of prematurity.

## Discussion

We aimed to determine the proportion of infants who did not complete scheduled ROP screening follow-up after referral to our tertiary service and to identify factors associated with interrupted follow-up. Loss to follow-up was common, with more than one in four infants failing to complete scheduled ROP follow-up. This level of attrition is clinically significant, as retinal findings may change after the initial examination and treatment-requiring disease may become evident only during subsequent reviews. The value of an ROP screening programme lies not only in identifying infants who require screening but also in ensuring that they remain under observation until retinal vascularisation is complete, disease has regressed, or treatment and post-treatment monitoring have been concluded. Our findings add to growing evidence that, in LMIC settings, improved neonatal survival has not always been matched by equally reliable systems for follow-up and continuity of care.^7,19,21^ South African reports have highlighted persistent weaknesses across the ROP pathway, including gaps in screening coverage, delayed review and poor retention in follow-up.^10,11,22^

The most robust association was observed between documented ROP at the first visit and LTFU, which persisted on the primary adjusted and in sensitivity analysis. This is concerning, as infants with established retinal disease are those most in need of careful ongoing surveillance. Variation in ROP prevalence and incomplete screening in other South African units has been described^23^, where infants with treatment-requiring ROP have also been shown to be susceptible to LTFU.^15^ Retinopathy of prematurity screening is resource intensive and exists specifically to detect and treat disease before childhood blindness develops. Loss to follow-up in infants with detected ROP extending even to those with severe and treatment-requiring disease is especially worrying, and directly undermines the objective of screening. The association is also unlikely to be an artefact of ascertainment. Retinopathy of prematurity recorded at any visit is more likely to be detected in infants who attend more examinations, so any such bias would tend to weaken rather than strengthen the observed association with LTFU. Studies from high-income countries (HICs) have shown that outpatient attendance is influenced by a mix of clinical risk, socioeconomic disadvantage, maternal and household factors, and the complexity of care after discharge.^16,18,20^ In the present setting, the pattern appears somewhat different. Loss to follow-up was more frequent among infants with documented ROP, suggesting that those at greatest ophthalmic risk may be the same infants whose families bear the greatest practical burden. Repeated appointments, travel between facilities and the wider demands of caring for a medically fragile preterm infant may all contribute to interruptions in care.

Maternal HIV exposure was associated with LTFU in univariable analysis and in the sensitivity model that included it. This may reflect overlapping social, economic, and health system vulnerabilities rather than a direct biological effect, and it should be interpreted cautiously given the substantial proportion of infants without recorded HIV exposure status. In contrast to the clear ROP finding, referral facility was not independently associated with LTFU, which is consistent with the non-significant distribution of LTFU across facilities in the univariable analysis. Facility level patterns should therefore be treated as hypotheses for local pathway audit rather than as demonstrated differences. Plausible service level influences on follow-up, such as discharge preparation, caregiver counselling, booking processes, transport arrangements, accuracy of contact details and communication between neonatal and ophthalmic teams, were not directly measured in this study and are best regarded as candidate targets for quality improvement work and local audit.^14,19^

Lower birth weight and lower gestational age were associated with LTFU in the unadjusted analysis, although these associations were attenuated after adjustment. Infants who did not complete follow-up had a lower mean birth weight and gestational age than those who remained in care, and a greater proportion had been born at 28 weeks or earlier or weighed less than 1000 g at birth. This is clinically plausible, as the smallest and most preterm infants often require longer admissions, more frequent ophthalmic review and ongoing management of multiple neonatal complications.^23,24^ For families, this often means more demanding post-discharge care, greater financial and logistical strain and a higher likelihood that ophthalmic follow-up will be interrupted. Similar associations between clinical vulnerability and incomplete follow-up have been described elsewhere, with many studies highlighting the role of social and structural barriers.^16,18,20^

Our findings are also consistent with international evidence indicating that outpatient ROP follow-up is shaped by more than clinical status alone. Studies from the United States have shown that attendance may be influenced by social determinants of health, area-level deprivation, and other socioeconomic barriers, in addition to medical risk.^18,25^ Other reports have shown that infants discharged from neonatal units while still requiring serial retinal examinations remain vulnerable to delayed attendance or complete LTFU.^16,26^ Although the data available for our study did not include direct measures of caregiver education, household income, transport access, mobile phone availability or reminder systems, these factors are likely to be relevant in the South African public-sector setting. The findings are best understood as reflecting an interaction between clinical risk, caregiver burden and fragmentation within the health system.

Our findings have practical implications for service delivery. Retinopathy of prematurity follow-up should be treated as a shared neonatal and ophthalmic pathway rather than a one-off outpatient visit. Attention is needed at discharge, when infants may leave the hospital while still requiring repeated retinal examinations. Clear documentation of the next appointment, careful counselling of caregivers, confirmation of contact details and systems for reminders and tracing missed visits are likely to strengthen continuity of care. Referral sites with higher attrition may benefit from targeted quality-improvement efforts focused on discharge processes, appointment scheduling, inter-team communication and accountability for missed follow-up. In settings where specialist access remains limited, teleophthalmology and related service innovations may offer additional opportunities to strengthen continuity and extend coverage.

### Strengths and limitations

One of the main strengths of this study is the large number of infants included over several years within a real-world tertiary referral service. This allowed follow-up patterns to be examined under routine clinical conditions rather than within a tightly controlled research setting. The use of both referral documentation and EMR data increases the practical value of the findings for local service improvement. The analysis also distinguished baseline characteristics from variables determined during follow-up, which avoids the circular interpretation that arises when post-baseline measures such as gestational age at last visit are treated as predictors.

Several limitations should be acknowledged. The retrospective design depended on the completeness and accuracy of routine clinical records, and some variables contained missing data. Infant HIV PCR status was recorded for only 171 infants and was excluded from the adjusted model, while maternal HIV exposure status was missing for a substantial minority and was therefore examined only in a sensitivity model. The definition of LTFU as non-attendance after the initial referral visit means that infants who never attended any visit after referral are not captured, so the true burden of disengagement is likely underestimated. Outcome misclassification is possible, as only IALCH EMR data were available. Follow-up occurring at other facilities could not be fully excluded, and infants who died, transferred care or remained in active follow-up at the database closing date were classified according to their attendance records rather than separately adjudicated. The timing of ROP ascertainment (any examination during the screening episode) means that detection depends partly on the number of attended examinations, although this would tend to bias the ROP LTFU association towards the null. Estimates for sparse categories were imprecise; the effective sample size of complete case models was reduced by missing data, and moderate collinearity was present among the correlated birth weight, birth gestational age and gestational age at first visit terms

Important contextual factors, such as caregiver education, household socioeconomic circumstances, transport access, distance to the facility, telephone access and reminder systems, were unavailable for analysis. These factors are likely highly relevant to follow-up attendance, and their absence limits how fully the observed associations can be explained. As our study was conducted at a single tertiary hospital, the findings may not be fully generalisable to all South African settings, although they are likely to be relevant to similar referral pathways in the public sector. Infants referred outside standard weight and gestational age criteria for other risk factors were not separately characterised.

### Implications and recommendations

Retinopathy of prematurity follow-up should be understood as a longitudinal care process shared by neonatal and ophthalmic services. Practical priorities include documenting the next appointment before discharge, ensuring that caregivers receive clear counselling about the risk of delayed disease progression, confirming contact and transport details, and strengthening reminder and tracing systems for missed appointments. Attention should be given to infants with documented ROP and to referral sites with higher attrition, as these groups appear especially vulnerable to interrupted care. Facility-level quality-improvement initiatives may help identify local barriers in referral preparation, appointment scheduling and communication between teams. Neonatal clinic follow-up proforma should include ROP screening retention as part of the checklist, as those lost to follow-up may still be attending neonatal clinic follow-up, and this creates an opportunity for reintegration into the ROP screening. Where specialist capacity is limited, teleophthalmology-supported screening and more structured referral coordination may provide additional value.

## Conclusion

Continuity of care is central to the success of ROP services in KwaZulu-Natal. More than one in four referred infants did not complete scheduled screening follow-up, and infants with documented ROP, who are among those most in need of surveillance, were the most vulnerable to interrupted care. Strengthening follow-up pathways will require coordinated action across neonatal and ophthalmic services, with particular attention to discharge planning, caregiver counselling, appointment systems, tracing of missed visits and communication between facilities. A more consistent and integrated approach to follow-up is likely to be essential in reducing avoidable visual impairment among preterm infants at risk of ROP.
